# Endocrine Disruptors in Pregnancy: Effects on Mothers and Fetuses—A Review

**DOI:** 10.3390/jcm13185549

**Published:** 2024-09-19

**Authors:** Rima Hajjar, Sana Hatoum, Serge Mattar, Gaby Moawad, Jean Marc Ayoubi, Anis Feki, Labib Ghulmiyyah

**Affiliations:** 1 Department of Obstetrics, Gynecology and Reproductive Sciences, University of Miami Miller School of Medicine, Miami, FL 33136, USA; hajjar.rima93@gmail.com; 2 Foundation for Research and Education Excellence, Vestavia, AL 35243, USA; sanahatoum@gmail.com; 3 Fertility & IVF Clinic, Dubai P.O. Box 72960, United Arab Emirates; 4 Department of Obstetrics and Gynecology, The George Washington University Hospital, Washington, DC 20037, USA; 5 Department of Obstetrics and Gynecology and Reproductive Medicine, Hôpital Foch-Faculté de Médecine, Suresnes, 92150 Paris, France; jm.ayoubi@hopital-foch.com; 6 Department of Obstetrics and Gynecology and Reproductive Medicine, HFR-Hopital Fribourgeois, Chemin des Pensionnats 2-6, 1708 Fribourg, Switzerland; anis.feki@hfr.ch; 7 Women’s Specialty Care of Florida, Pediatrix Medical Group, Fort Lauderdale, FL 33316, USA

**Keywords:** endocrine disruptors, bisphenol A, phthalic acids, organophosphates, fluorocarbons, environmental exposure, pregnancy, fetal development

## Abstract

**Background/Objectives**: Endocrine disruptors are ubiquitous agents in the environment and are present in everyday consumer products. These agents can interfere with the endocrine system, and subsequently the reproductive system, especially in pregnancy. An increasing number of studies have been conducted to discover and describe the health effects of these agents on humans, including pregnant women, their fetuses, and the placenta. This review discusses prenatal exposure to various endocrine disruptors, focusing on bisphenols, phthalates, organophosphates, and perfluoroalkyl substances, and their effects on pregnancy and fetal development. **Methods**: We reviewed the literature via the PubMed and EBSCO databases and included the most relevant studies. **Results**: Our findings revealed that several negative health outcomes were linked to endocrine disruptors. However, despite the seriousness of this topic and the abundance of research on these agents, it remains challenging to draw strong conclusions about their effects from the available studies. This does not allow for strong, universal guidelines and might result in poor patient counseling and heterogeneous approaches to regulating endocrine disruptors. **Conclusions**: The seriousness of this matter calls for urgent efforts, and more studies are needed in this realm, to protect pregnant patients, and ultimately, in the long term, society.

## 1. Introduction

The World Health Organization (WHO) defines an endocrine disruptor (ED) as “an exogenous substance or mixture that alters function(s) of the endocrine system and consequently causes adverse health effects in an intact organism, or its progeny, or (sub) populations.” [1] These agents are ubiquitous in the environment and found in numerous everyday consumer products [1,2,3]. Exposure can also occur through contaminated food, contaminated groundwater, and combustion sources [1].

EDs often enter the environment via agricultural and industrial practices [2,3]. Agricultural EDs include phytoestrogens, persistent organic pollutants (POPs), and the herbicide glyphosate. While phytoestrogens (such as daidzein and genistein) are naturally found in soybeans and other legumes, POPs such as dichlorodiphenyltrichloroethane (DDT) and its metabolite dichlorodiphenyldichloroethylene (DDE) are synthetic Eds, for which the exposure sources include pesticides, waste burning, and paper bleaching [2,3]. Bisphenols, including bisphenol A (BPA), bisphenol S (BPS), bisphenol F, and bisphenol AF (BPAF), and high-molecular-weight phthalates, such as bis (2-ethylhexyl) phthalate (DEHP) and its metabolite mono-2-ethylhexyl phthalate (MEHP), are often used in plastics, food packaging, and dental sealants [2,3], while low-molecular-weight phthalates are often used in cosmetics and personal hygiene products [3]. Polycyclic aromatic hydrocarbons include polybrominated diphenylethers (PBDEs) and polychlorinated biphenyls (PCBs), and are commonly used in electronics, furniture, hydraulic fluids, and flame retardants for the insulation of electrical installations [2,3]. Parabens are used as preservatives in cosmetics [2], and PFAS are synthetic EDs found in polishes, paints, and non-stick cookware [3].

The human endocrine system is quite complex in its interaction with multiple organ systems, especially the reproductive system. EDs can interfere with these interactions, leading to dysregulation. A developmental origin of health and disease theory, namely the “Barker hypothesis”, as pioneered by epidemiologist Dr. David Barker, suggests a link between adult metabolic disorders and the preconceptual, fetal, and early infant phases of life [4]. This groundbreaking theory suggested that diet, lifestyle, and environmental insults can impact the maternal–fetal–placental unit and ultimately lead to disease in adult life. Similarly, it is believed that EDs can induce epigenetic modifications in fetal cells in utero. Some EDs have also been described as leading causes of reproductive disorders, impacting fertility and causing gamete anomalies during fetal development [3]. Pregnancy thus represents a critical period, yet data on ED exposure in pregnancy appear to be scarce. Most studies have focused on accidentally highly exposed groups and external environmental sources of contamination, such as air, food, and water, rather than internal exposure (of blood and tissue) [1], with limited in vivo studies. In addition, most studies on ED exposure in pregnancy have been divergent, examining different outcomes at different points in time.

In light of the growing awareness and concern, stakeholders have established regulations and passed legislation to identify and control the use of such agents. Despite the tremendous disease cost due to EDs, and the fact that several health, medical, and scientific organizations have released statements on their harmful effects, current regulatory agencies rarely hold manufacturers responsible for the consequences of these agents [5].

Our objective is to create a list of EDs commonly encountered in pregnancy and their effects on pregnancy and fetal development, discussing the available research and its limitations. We conducted a thorough literature review using the PubMed and EBSCO databases, with a focus on studies published within the last 12 years that examine the effects of bisphenols, phthalates, organophosphates, and perfluoroalkyl substances on pregnancy and fetal outcomes. The literature review was performed using various combinations of keywords, including endocrine disruptors, bisphenols, phthalates, organophosphates, perfluoroalkyl substances, pregnancy, maternal exposure, environmental exposure, fetal exposure, fetus, fetal development, and fetal outcomes.

## 2. Mechanism of Action

The prenatal period is a very sensitive window for fetal development. Any perturbation in the maternal endocrine system or placental function can lead to derangement in fetal growth and development. At a molecular level, EDs can alter gene expression by interacting directly with a family of nuclear hormone receptors (NHRs) that function as transcription regulators to either activate or repress gene function. A similar transcription factor that also serves as a target for EDs is the aryl hydrocarbon receptor (AhR), which regulates the expression of several genes, including the cytochrome P450 (CYP)-1 gene family members [6]. The biological mechanisms and molecular signaling pathways are quite complex and remain subjects of growing interest and research.

The placenta regulates multiple endocrine, immunological, and physiological processes throughout pregnancy, but it is not always an effective barrier against EDs [6]. EDs including PBDEs, BPA, and PCBs can potentially pass from the mother to the fetus through the placenta, and have been detected in the placenta [6,7]. Aside from crossing the placenta, entering fetal circulation, and accumulating in fetal tissue, EDs can disrupt placental growth and function. The susceptibility of the placenta to EDs is largely due to the extensive expression of hormone receptors in the placenta and the ability of EDs to alter hormonal equilibrium, either by binding to these receptors and hormone transport proteins or by interfering with the degradation and synthesis of endogenous hormones [7]. For instance, PBDE and PAH levels in umbilical cord blood correlate with the expression of IGF-1 and IGFBP-3 mRNA levels in the placenta [8]. Since EDs can alter, mimic, or disrupt the function of gestational hormones, pregnancy remains especially sensitive to their actions [9]. In addition, the lack of placental and fetal enzymes as weaponry against EDs [7] makes the fetus highly vulnerable to any alterations that can disrupt fetoplacental homeostasis. Thus, the effects of EDs on the fetoplacental unit should not be underestimated.

Finally, the uterus can also be affected by exposure to EDs. Studies have shown that certain Eds, such as BPA, can arrest endothelial proliferation and decrease vascular endothelial growth factor (VEGF) mRNA expression, thus interfering with implantation [2].

## 3. Common Endocrine Disruptors: Sources and Effects on Pregnancy and Fetal Development

### 3.1. Bisphenol A

#### 3.1.1. Sources of Exposure

Bisphenols are released mainly from polycarbonates and epoxy resins and include BPA, BPS, bisphenol F, bisphenol M, and BPAF. Bisphenols are often used in food packaging, plastic dinnerware, dental sealants, and thermal receipts [2,3,9]. Notably, over 90% of overall BPA exposure is thought to be through diet [10], and BPA was banned by the EU Commission Regulation from the manufacture of infant feeding bottles in 2011 [11]. While BPA intake has been shown to vary from one country to another, pregnant women have been noted to have significantly higher BPA intakes than the general population [12]. Several studies have shown that BPA is transported through the placenta and can accumulate in the placental trophoblast and affect placental growth.

#### 3.1.2. Postnatal Endocrinological Outcomes

Several studies have examined the effect of prenatal BPA exposure on the development of diabetes, obesity, and cardiovascular disease in adulthood. Animal studies have been conflicting; some suggested that BPA might have an obesogenic effect while others showed no association between BPA and BMI, with some even showing an inverse correlation between BPA exposure and BMI [13]. The reason behind these discrepancies is unclear but might be explained by differences in the methodologies used to administer BPA, the doses and timing of administration, the types of species involved, and the sample sizes. It has been suggested that BPA exposure at different periods can affect β-cell pancreatic function and lead to differences in the quantity and quality of β-cells, ultimately leading to decreased insulin production. Insulin resistance also occurs, leading to increased adiposity and decreased glucose tolerance [14]. In vitro and in vivo studies have shown that BPA exposure can induce adipogenesis and triglyceride accumulation in mice and inhibit the release of adiponectin, a hormone present in adipocytes that is believed to protect against insulin resistance and metabolic syndrome [15].

Few studies have examined BPA exposure in humans, and care must be taken when extrapolating from animal studies due to the developmental difference between species [14]. For example, pancreatic development in rodents occurs both in the prenatal and postnatal period, whereas in humans the bulk of the development occurs in the prenatal period. Several human studies showed an association between BPA exposure in childhood and adulthood, and obesity [16]. However, most were cross-sectional and did not focus on the prenatal window of exposure. Only three studies (two US cohorts and one European) evaluated BPA exposure in utero by measuring maternal creatinine-adjusted urinary BPA concentrations and childhood-related obesity outcomes [13,15,16]. Both the California and Cincinnati cohorts showed that, contrary to findings from animal studies, prenatal BPA exposure in humans was not associated with significant changes in BMI. In fact, both studies found that a high maternal urinary BPA was associated with a lower BMI in childhood amongst girls; however, the association was not significant. The Spanish cohort study found that maternal urinary BPA concentrations during pregnancy were associated with increased childhood BMI and waist circumference, manifesting at four years of age. Differences in outcomes between these cohorts could be due to confounding variables, misclassification of BPA exposure, and selection bias.

The effect of maternal BPA exposure during pregnancy on fetal thyroid function has been investigated in the Center for the Health Assessment of Mothers and Children of Salinas (CHAMACOS) study, and an inverse association was found between maternal BPA concentration and total T4 levels, but not free T4, specifically in male offspring. The association appears to be stronger in the third trimester, which may suggest either a transient effect or, alternatively, a window of susceptibility and concern [17].

The CHAMACOS cohort was also used to study the effects of phthalates and BPA on pubertal development and revealed that high-molecular-weight phthalates and BPA were associated with later-onset puberty in girls and earlier-onset puberty in boys [18].

The Infant Development and the Environment Study (TIDES) is another cohort study designed to evaluate the effects of BPA on the reproductive development of female offspring by using anogenital distance as a biomarker of the fetal hormonal milieu and a measure of reproductive toxicity. Higher first-trimester BPA exposure was associated with significantly shorter anogenital distance in daughters, suggesting that BPA may impact reproductive development [19].

#### 3.1.3. Postnatal Cardiovascular Outcomes

In a recent prospective cohort study of 935 mother–child pairs, Blaauwendraad et al. hypothesized that maternal exposure to bisphenols during different trimesters of pregnancy can affect fetal vasculature and impair arterial health [20]. However, a higher maternal urinary total bisphenol level, specifically BPA concentrations, was associated with lower carotid intima-media thickness. This was contradictory to prior studies that showed a positive correlation between higher BPA exposure and carotid intima-media thickness. Notably, these studies were conducted in adults exposed to bisphenols, whereas Blaauwendraad et al.’s study is the only study to date conducted in offspring exposed to bisphenols prenatally. The discrepancy in the results could be explained by the differences in pathophysiology in the two populations. In adults, BPA exposure seems to increase oxidative stress, which is known to accelerate the formation of atherosclerotic plaques, whereas, in the fetal period, exposure could cause structural vascular adaptations rather than the development of plaques. The significance of a thinner carotid intima-media is still unclear and whether or not this could lead to weaker, more “vulnerable” vasculature remains under question.

#### 3.1.4. Postnatal Neurological Outcomes

Grohs et al. examined the effects of prenatal BPA exposure on brain microstructure in 98 mother–child pairs from the Alberta Pregnancy Outcomes and Nutrition (APrON) study in Canada [21]. The investigators found an association between maternal urinary BPA concentrations during the second trimester and poor development of the splenium and the right inferior longitudinal fasciculus, suggesting that prenatal exposure to higher BPA doses during the second trimester may result in less developed white matter in the inferior and posterior brain regions, as compared to children exposed to lower BPA doses. Interestingly, no association was found between postnatal urinary BPA and white matter development, suggesting that prenatal rather than postnatal BPA exposure may shape and organize brain development. Similar studies have supported this finding; however, this study was unique because investigators used MRI findings as quantitative effect biomarkers.

Numerous studies support the notion that early BPA exposure can impact childhood internalizing and externalizing behavior. Braun et al. concluded that maternal urinary BPA concentration during pregnancy was associated with some aspects of children’s behaviors at three years of age; specifically, poorer reciprocal social behaviors among all children, more internalizing and somatization behaviors in boys, and poorer working memory and planning/organizing abilities in boys [22]. Similarly, the Odense child cohort study revealed that prenatal BPA exposure, even in low concentrations, may increase the risk of Autism Spectrum Disorder (ASD) symptoms, which may predict later social abilities [23].

#### 3.1.5. Pregnancy Loss

After a thorough literature review, we identified only one study evaluating the effects of BPA exposure in the luteal phase and miscarriage. BPA was found to be associated with a shorter luteal phase but not associated with an increased time to pregnancy or early pregnancy loss. These findings are of particular interest because the samples were collected from 221 healthy women during a period (1982–1986) where BPA exposure was generally higher than it is nowadays [24].

#### 3.1.6. Preeclampsia

Two case-control studies found an association between maternal BPA concentrations and preeclampsia [25,26]. The case-control study led by Leclerc noted BPA accumulation in the placenta of preeclamptic women, but not the umbilical cord or peripheral maternal blood, suggesting that the UGT pathway, which is the main pathway for BPA metabolism, is altered more selectively in the placenta that in the liver of preeclamptic women, leading to BPA accumulation in the placenta rather than in peripheral blood [25]. The research group also suggested that BPA is believed to alter angiogenesis by impairing estrogen production through downregulation of aromatase activity, thus leading to preeclampsia and other complications. Other researchers have also suggested that elevated BPA concentrations can induce apoptosis of trophoblastic cells, which could explain the pathophysiology of preeclampsia.

#### 3.1.7. Preterm Birth

Two nested case-control studies, one in Boston and the other in Mexico City, evaluated the effect of prenatal maternal BPA exposure on preterm birth [27,28]. The Mexico City study group found an OR = 2.5 of having a preterm birth in relation to third trimester BPA concentration [28]. The Boston study group was not found to have a significant association between BPA concentration and preterm birth. However, when the investigators further stratified the results by type of preterm birth (spontaneous preterm birth, defined by the investigators as preterm birth resulting from preterm labor or premature rupture of membranes vs. placental etiology) and neonatal sex, they found a significant association between BPA levels in the third trimester and high rates of spontaneous preterm birth of female infants specifically [27]. These findings could be explained by the fact that BPA has been found to stimulate the production of pro-inflammatory cytokines, which could initiate an inflammatory cascade, to which female infants appear to be more susceptible. This cascade then leads to preterm labor or rupture of membranes. Interestingly, there was no association between BPA concentration and preterm delivery due to placental etiology (growth restriction and preeclampsia), despite previously proposed mechanisms of BPA-induced trophoblastic apoptosis and impairment of estrogen-mediated angiogenesis. Given the scarcity of data, these results should be interpreted with caution.

#### 3.1.8. Fetal Growth

Several nested case-control and cohort studies investigated the effect of BPA exposure on fetal growth with inconsistent results. Snjider et al. studied a cohort of 219 Dutch women and found that women exposed to higher BPA levels had lower growth rates for fetal weight and head circumference [29]. Another nested case-control study from China also concluded that urinary BPA concentrations in the third trimester were associated with an increased risk of low birth weight [30]. In contrast, a study from South Korea [31] and a French cohort study found positive associations between maternal BPA concentrations and fetal head circumference [32]. Several studies from the US and Spain did not report a significant association between BPA exposure and fetal growth [33,34].

The discrepancies between study results may stem from various factors, such as differences in BPA sample collection methods; some used a single urine sample, while others collected multiple samples throughout pregnancy trimesters. In addition, a lack of assessment of other confounding EDs in some studies could also influence outcomes.

### 3.2. Phthalates

#### 3.2.1. Sources of Exposure

Phthalates are chemicals, often referred to as “plasticizers”, that are present in everyday products [35,36]. High-molecular-weight phthalates are often used to synthesize flexible plastic used in food storage containers, medical equipment, and flooring, and exposure is often through ingestion, whereas low-molecular-weight phthalates are often used in personal care items (e.g., shampoos, lotions, and fragrances), with exposure usually occurring through dermal absorption or inhalation [35].

#### 3.2.2. Preterm Birth

In a recent systematic review and meta-analysis, Wang et al. included seven studies investigating the association between phthalates and preterm birth and found a positive association for several metabolites, including mono-n-butyl phthalate (MBP), sum of di-2-ethylhexyl phthalate (ΣDEHP), and mono 3-carboxypropyl phthalate (MCPP) [37]. A limitation of this meta-analysis is the lack of stratification of phthalate exposure by trimester. However, current data from several cohort studies, including Furgeson’s PROTECT study [38], a Boston cohort study [39], and the MIREC pan-Canadian cohort study [40] suggest that the relationship between preterm birth and phthalate exposure is dose-dependent and strongest in later gestation, particularly in the late second trimester and third trimester. Few studies have stratified the outcomes of prenatal phthalate exposure by fetal sex. In a recent study, Cathy et al. utilized the PROTECT cohort to further explore differences in these associations based on fetal sex and found a stronger selection for monoisobutyl phthalate (MiBP), monohydroxyisobutyl phthalate (MHiBP), and monocarboxynonyl phthalate (MCNP) with spontaneous preterm birth among women carrying a male, compared to a stronger selection of MBP and monohydroxybutyl phthalate (MHBP) for preterm birth for mothers carrying a female fetus [35]. Several studies have demonstrated an increase in biomarkers of oxidative stress and inflammation in women exposed to phthalates, which explains the early initiation of labor in these women [41,42].

#### 3.2.3. Preeclampsia

A recent systematic review and meta-analysis included 10 human epidemiological studies to investigate the relationship between phthalate exposure and blood pressure (BP) changes in pregnancy. The findings suggest that several metabolites, including MBP, monobenzyl phthalate (MBzP), and MEHP are associated with BP changes in pregnancy, and monoethyl phthalate (MEP) in particular was associated with hypertensive disorders of pregnancy [43]. Data from the Health Outcomes and Measures of the Environment Study (HOME) suggest that maternal urinary MBzP concentrations may be associated with increased diastolic BP and risk of pregnancy-induced hypertensive diseases [44]. The effects of phthalates on BP appear to extend to the postpartum period. Women participating in the Programming Research in Obesity, Growth, Environment, and Social Stressors (PROGRESS) longitudinal study were found to have elevated BPup to 72 months postpartum [45].

Several pathways were proposed to explain the effects of phthalates on BP, but the exact mechanism remains uncertain [43]. One pathway is the renin-angiotensin-aldosterone system, which can be inhibited by phthalates. Multiple studies have also shown that phthalates can increase oxidative stress, ultimately leading to an increase in circulating angiogenic factors linked to hypertensive disorders in pregnancy. More studies are emerging on the effects of phthalates on maternal thyroid function and how they could potentially indirectly impact BP throughout pregnancy.

#### 3.2.4. Gestational Diabetes

In a recent systematic review, phthalate exposure during pregnancy was associated with poor glycemic control. Additionally, three out of five human studies revealed a positive association between several phthalates and gestational diabetes mellitus [46].

#### 3.2.5. Thyroid Function

Several epidemiological studies have investigated the effect of phthalates on maternal thyroid function and demonstrated that some phthalate metabolites can potentially alter maternal thyroid function; however, the direction and magnitude of this effect are still uncertain. Huang et al. observed a significant association between phthalates and plasma-free and total T4. In contrast, other studies showed no association between phthalate metabolites and free and total T4 [47]. These results also conflict with studies on non-pregnant women and adult men. Kuo et al. and Johns et al. both report an inverse association between phthalate metabolites and TSH [48,49]. These studies have not assessed the level of thyroid antibodies.

During the first trimester, the fetus relies exclusively on maternal thyroid function, and alteration in maternal thyroid function can potentially affect the fetus negatively. Several mechanisms were proposed to explain how phthalates can potentially alter maternal thyroid function by affecting the hypothalamic–pituitary–thyroid axis and/or interfering with thyroid biosynthesis and biotransport, peripheral conversion, as well as receptor function [48].

#### 3.2.6. Fetal Growth

Studies have been inconsistent regarding the effects of phthalate and fetal growth restriction or low birth weight. The inconsistencies appear to arise from the heterogeneity of these studies and limitations that arise when comparing the effects of different single phthalates to phthalate mixtures. The data suggest that the effects of phthalates on birth weight may be skewed by gender and/or gestational age at the time; however, more data are needed. A recent study on a cohort in Wuhan, China revealed trimester-specific and sex-specific effects of prenatal exposure to DEHP and its metabolites. For example, the study revealed significant negative relationships between maternal urinary DEHP levels and fetal growth, and significant positive associations of some DEHP metabolites with average postnatal weight and BMI z-scores, among male offspring. While some DEHP metabolites during the third trimester were significantly related to weight gain rates from 6 to 12 months of age among boys, DEHP levels during the second trimester were positively related to weight gain rates at 6, 12, and 24 months old among boys. Among girls, however, a significant negative relationship between DEHP during the first trimester and weight gain rates was reported, while no significant associations between DEHP and its metabolites and weight gain rates at 12 and 24 months were observed. [50]. Another study on a Japanese cohort reported that DEHP exposure causes reduced fetal weight and crown-rump length, in a gender-independent manner [51]. A third study on a cohort from Boston also observed an inverse association between urinary ∑DEHP metabolites levels and fetal growth [52]. It is hypothesized that one of the mechanisms that leads to fetal growth restriction is the effect of phthalates on placental weight, which ultimately leads to placental insufficiency and intrauterine growth restriction [53].

#### 3.2.7. Prenatal and Postnatal Neurodevelopmental Outcomes

Some studies on postnatal phthalate exposure have demonstrated a decline in IQ scores [36,54], while some showed no association between this exposure and psychomotor development [55]. The mechanism explaining the results of the former group of studies remains uncertain, but one proposed mechanism is the alteration of thyroid function essential to neurodevelopment in utero. Other proposed mechanisms are a decrease in the number of midbrain dopaminergic neurons, tyrosine hydroxylase biosynthetic activity, and tyrosine hydroxylase immunoreactivity [36,54]. Studies on prenatal exposure to phthalates on neurocognitive development have remained inconsistent; while some found that exposure was not associated with children’s IQ scores [36] or neurodevelopment [56] (where sex plays the role of an effect modifier [56]), others showed an inverse relationship between this exposure and a child’s motor development [55], decreases in psychomotor development, and with increased odds of psychomotor delay [57].

A few studies, including the MARBLES study, have investigated the effect of prenatal phthalate exposure on autism. In this cohort, 14 metabolites of eight phthalates in 636 multiple maternal urine samples were collected during the second and third trimesters of pregnancy from 201 mother–child pairs, and at three years old, children were clinically assessed for ASD. The study concluded that there is no increased risk of ASD in children exposed to phthalates in mid-to-late pregnancy [58]. In a more recent prospective study on a Canadian cohort of 2001 pregnant women, higher gestational concentrations of some phthalate metabolites in the first trimester were associated with higher scores for autistic traits in boys, but not girls; however, it appears that these effects can be mitigated by folic acid supplementation [59].

### 3.3. Organophosphates

#### 3.3.1. Sources of Exposure

Organophosphates are often found in pesticides (herbicides and insecticides), chemical weapons, plastic packaging, flame retardants, and lubricants [60,61,62]. Exposure is predominantly through dermal absorption or ingestion, and ingestion of crops sprayed with organophosphate pesticides contributes largely to organophosphate poisoning in humans [60]. Accidental intoxication and suicide attempts with organophosphate pesticides are also a serious concern [61].

#### 3.3.2. Neurodevelopment

The primary target of organophosphates is acetylcholinesterase (AChE), which degrades the neurotransmitter acetylcholine. Through poisoning, excess acetylcholine builds up and works on several effector regions, mainly the neuromuscular junctions, skeletal nerve–muscle junctions, the central nervous system, and autonomic ganglia [60]. Several studies have focused on organophosphate exposure and neurobehavioral development. In the CHAMACOS study, the investigators describe an inverse relationship between the agricultural use of organophosphate pesticides within 1 km of maternal residences during pregnancy and cognitive development in children at seven years of age [63]. In a Chinese prospective cohort study, Wang et al. used the Bayley scale, mental development index (MDI), and psychomotor development index (PDI) to evaluate the effect of prenatal exposure to organophosphates on cognition, language, and social development. The investigators concluded that prenatal exposure to organophosphates, especially in the first trimester, was associated with lower Bayley scores in children, particularly in boys [64]. A systematic review that included 13 studies from low- to middle-income countries, however, found inconsistent associations between pregnancy exposure to organophosphates, pyrethroids, and carbamates and child development up to seven years of age [65]. Similarly, the French PELAGIE cohort study and the Generation R study found no significant associations between IQ scores and prenatal exposure to organophosphates [66,67].

The New York Mount Sinai cohort study concluded that prenatal organophosphate metabolites in urine were associated with an increasing number of abnormal primitive reflexes in neonates, as evaluated by the Brazelton Neonatal Behavioral Assessment Scale [68]. Similarly, the CHAMACOS cohort study in California concluded that prenatal exposure to organophosphates was associated with poorer neonatal reflexes, attention skills, and intellectual development [69]. These findings were also comparable to the Shenyang Chinese cohort study, which noted lower Neonatal Behavioral Neurological Assessment (NBNA) scores in neonates exposed to organophosphates [70].

Several studies have noted an association between organophosphate exposure prenatally and the development of ASD, including the CHARGE study which collected data from mothers who lived within 1.5 km (just under one mile) of an agricultural pesticide application [71], and the CHAMACOS study [72]. Schmidt et al. also described the attenuating effect of folic acid supplementation in the first month of pregnancy on ASD in women exposed to organophosphates [73].

#### 3.3.3. Fetal Growth

The largest study on organophosphate effects on fetal growth included 1235 women from the CHAMACOS, HOME, Columbia, and Mount Sinai birth cohorts. Key differences between the cohorts included enrollment years, gestational age at which urine samples were collected, the number of urine samples collected, and race/ethnic composition. The investigators correlated prenatal exposure among black women with decreased infant size at birth but found no evidence of lower birth weight, birth length, or head circumference among whites or Hispanics [74]. The second largest study, conducted on a cohort of 858 women from Denmark, did not find an association between organophosphate exposure measured at 28 weeks of gestation and birth weight, birth length, or abdominal or head circumference at delivery [75]. The third largest study was conducted on the Generation R Rotterdam cohort and found that organophosphate exposure was associated with decreased fetal weight and length measured during mid-pregnancy, but not at delivery [76].

### 3.4. Perfluoroalkyl Substances

#### 3.4.1. Sources of Exposure

Perfluoroalkyl substances (PFAS) are commonly found in non-stick cookware, food packaging, shampoos, lubricants, and carpets [77,78,79]. They pass through the food chain via ingestion of contaminated food and beverages, the main source of direct exposure to PFAS [79]. This large group of chemicals includes perfluoroalkyl carboxylic acids (PFCAs), perfluorooctanoic acid (PFOA), perfluoroalkyl sulfonic acids (PFSAs), perfluorohexane sulfonic acid (PFHxS), and perfluorooctane sulfonate (PFOS) [77,79]. Not only are PFAS ubiquitous, but they are also extremely stable, and most are resistant to degradation, resulting in increasing accumulation in the environment [77,78,79].

#### 3.4.2. Miscarriage

In a case-control study, strongly significant associations were reported between serum concentrations of PFAS (perfluorodecanoic acid (PFDA) and, especially, perfluorononanoic acid (PFNA)) and miscarriage, and almost significant associations with PFHxS exposure, while several studies reported no consistent link to miscarriage and stillbirths in a population with high PFOA exposure [79].

#### 3.4.3. Thyroid Function

A recent systematic review included 12 studies assessing the effect of prenatal exposure to PFAS on thyroid function [80], aiming to determine whether a link exists between PFAS exposure prenatally, thyroid function, and ASD. In this review, Shin et al. hypothesized that thyroid dysfunction following PFAS exposure can affect fetal brain development. One out of the 12 studies found no association between PFAS exposure and thyroid dysfunction, while the rest showed altered thyroid hormone levels in either maternal blood or the umbilical cord after PFAS exposure. The direction and the magnitude of the association remained inconsistent throughout the studies. In addition, most included studies failed to measure important biomarkers that might affect maternal thyroid function, such as iodine status or thyroid antibodies [80].

#### 3.4.4. Obesity

Three recent meta-analyses evaluated the effects of prenatal PFAS exposure on the development of childhood obesity. Fragione et al. concluded that prenatal PFAS (except for PFHxS) exposure may increase the risk of childhood obesity, as measured by BMI; however, these findings were not statistically significant [81]. These findings echoed those of Stratakis et al., who found similar positive but non-statistically significant associations [82]. Similarly, Frigerio et al.’s comprehensive systematic review suggests mostly positive possible associations between prenatal exposure to some PFAS and childhood BMI [83].

One proposed mechanism by which PFAS appears to alter childhood adiposity is via activation of PPARα signaling pathways involved in modulating lipid and glucose metabolism and the differentiation of adipocytes. Another proposed mechanism is via alteration of the hypothalamic–pituitary–thyroid axis [81].

#### 3.4.5. Fetal Growth

Several studies have suggested an association between PFAS exposure and low birth weight. In their meta-analysis, Gui et al. included 46 studies and concluded that certain types of PFAS are increasingly proven to partly reduce physical measures such as birth weight, birth length, and head circumference, and increase the incidence of adverse birth outcomes such as preterm birth, low birth weight, and small size for gestational age [84].

The modulating effects of folate supplementation on the association between PFAS and fetal growth have also been a subject of interest. In a recent prospective prebirth cohort study that included 1400 mother–singleton pairs, Zhang et al. concluded that higher early pregnancy PFAS concentrations were associated with lower birth weight only among mothers whose early pregnancy dietary folate intake or plasma folate levels were below the 25th percentile [85].

#### 3.4.6. Neurobehavioral Disorders

Several studies have been published on the association between prenatal PFAS exposure and neurobehavioral disorders, particularly attention deficit disorders (ADHD), autism, and behavioral disorders. The results of these studies remain inconclusive. Forns et al. included nine European population-based studies encompassing over 4826 mother-child pairs and concluded there was no increased prevalence of ADHD in association with either exposure to PFOS or PFOA. However, the stratified analyses of this study suggest that there may be an increased ADHD prevalence in association with PFAS exposure in girls, children from nulliparous women, and children from low-educated mothers. Studies included in this meta-analysis did not exclusively focus on prenatal exposure to PFOS and PFOA but also included studies with concentrations of PFOS and PFOA measured in maternal serum/plasma during pregnancy, or in breast milk, with different timings of sample collections in each cohort [86].

In a recent meta-analysis focused exclusively on prenatal exposure to PFAS and including 11 studies encompassing 8493 participants, Yao et al. concluded that PFOA and PFOS exposure during pregnancy might be associated with ADHD in offspring, while prenatal PFOS and PFNA exposure might be associated with ASD in offspring [87]. In the PELAGIE mother–child cohort study, prenatal exposure to nine PFAS was measured from concentrations in cord serum samples and behavior was assessed at age 12 years of age in 444 children. The investigators’ findings suggest that PFAS exposure prenatally is not only associated with externalizing behaviors but also with internalizing behaviors such as general anxiety and major depressive disorder [88].

Table 1 summarizes the key findings discussed in our review.

## 4. Prevention

Recent years have seen a rise in animal and human studies on prenatal ED exposure and its effects on maternal and fetal health. Despite abundant research, results remain heterogeneous and difficult to interpret and draw precise causal–effect conclusions. Studies also remain insufficiently powered to detect significant results. Challenges to research include difficulty in quantifying exposure, comparing single compounds vs. mixtures of compounds, differentiating exposure by pregnancy trimester, and assessing longitudinal outcomes to account for the time lag between exposure and manifestation of disease while still accounting for potential confounders. Exploring epigenetic and dose-related effects further complicates study design and calls for new research designs with more robust methods.

Despite these limitations and the absence of causal evidence, The Royal College of Obstetricians and Gynecologists stresses the importance of informing mothers of the sources and routes of exposure, the potential harmful fetal and maternal effects, and the importance of minimizing exposure [89]. Also, recognizing EDs as an emergent global health hazard, especially to pregnant women, the American College of Obstetricians and Gynecologists and the American Society for Reproductive Medicine have both joined the American Academy of Pediatrics and numerous other health professional organizations in calling for timely action to identify and reduce exposure to toxic environmental agents while addressing the consequences of such exposure [90]. In addition, the International Federation of Gynecology and Obstetrics (FIGO) also published an opinion on the reproductive health impacts of exposure to toxic environmental chemicals and stressed the role of reproductive health professionals in prioritizing preventing exposure to environmental chemicals everywhere [91]. These recommendations emphasize the importance of training obstetricians to recognize exposure sources and identify at-risk women to better counsel these patients.

## 5. Conclusions

In this review, we summarize the most important endocrine disruptors and their effects on the fetus, mother, and pregnancy following in utero exposure. The prenatal period represents a sensitive time window that should not be overlooked, and the potentially harmful effects of EDs should not be understated. Despite the seriousness of this matter, it remains challenging to draw strong conclusions about Eds’ effects from the available literature. We urge providers to be vigilant and dedicate more time to counseling pregnant women on EDs using the evidence and guidelines that are currently available, yet we stress the importance of conducting more research on EDs, with meticulous methodologies and careful sampling of subjects, in an attempt to produce a reliable body of evidence for future guidelines.

## Figures and Tables

**Table 1:** Summary of the Key Findings on the Effects of Endocrine Disruptors on Mother and Fetus.

Endocrine Disruptor	Key Findings
**Bisphenols** (including **BPA**, bisphenol S, bisphenol F, bisphenol M, and bisphenol AF)	***Pregnancy course and fetal outcomes:*** conflicting findings, with sex differences - BPA associated with a shorter luteal phase but not with increased time to pregnancy or early pregnancy loss [24] - Association between maternal BPA concentration and preeclampsia [25,26] - Prenatal BPA exposure increased odds of preterm birth [28] and rates of spontaneous preterm birth of female infants [27] - Conflicting outcomes about effect of BPA exposure on fetal growth [29,30,31,32,33,34] ***Neurocognitive outcomes:*** mostly negative effects, with sex differences Prenatal BPA exposure associated with - Poor development of the splenium and in the right inferior longitudinal fasciculus (but no association between postnatal urinary BPA and white matter development) [21] - Poorer reciprocal social behaviors among all children, more internalizing and somatization behaviors in boys, and poorer working memory and planning/organizing abilities in boys [22] - Possible increased risk of ASD symptoms (even in low concentrations) [23] ***Endocrine effects:*** conflicting findings, with sex differences - BPA exposure in childhood and adulthood associated with obesity [16], but in utero BPA exposure had conflicting outcomes [13,15,16] - Inverse association between maternal BPA concentration and total T4, but not free T4, specifically in male offspring, with stronger association in the third trimester [17] - Association with later puberty in girls and earlier puberty in boys for BPA and high-molecular-weight phthalates [18] ***Miscellaneous*** - Higher first-trimester BPA exposure associated with significantly shorter anogenital distance in daughters [19] - Higher maternal urinary total bisphenol, specifically BPA concentrations, associated with lower carotid intima-media thickness, contrary to studies on adult exposure [20]
**Phthalates**	***Pregnancy course****:* mostly negative effects - Several metabolites associated with BP changes in pregnancy (particularly MEP associated with hypertensive disorders of pregnancy) [43] - MBzP concentrations may be associated with increased diastolic BP and risk of pregnancy-induced hypertensive diseases [44] - Phthalate BP effects extend postpartum (elevated BP up to 72 months postpartum) [45] - Phthalate exposure during pregnancy associated with poor glycemic control, three of five studies showed positive association between several phthalates and GDM [46] - Some phthalate metabolites can potentially alter maternal thyroid function with uncertain direction and magnitude of this effect—significant association between phthalates and plasma-free and total T4, conflicting with other studies that showed no association [47] and inverse association between phthalate metabolites and TSH [48,49] ***Preterm birth:*** mostly negative effects, with sex differences - Positive association between phthalates and preterm birth for several metabolites [37] - Dose-dependent relationship between preterm birth and phthalate exposure, strongest in later gestation [38,39,40] - Stronger selection for MiBP, MHiBP, and MCNP with spontaneous preterm birth among women carrying a male fetus compared to a stronger selection of MBP and MHBP for preterm birth for mothers carrying a female fetus [35] - Increased biomarkers of oxidative stress and inflammation in women exposed to phthalates, explaining early initiation of labor [41,42] ***Fetal outcomes:*** conflicting findings with mostly negative effects, with sex differences - Significant negative relationships between maternal urinary DEHP levels and fetal growth, and significant positive associations of some DEHP metabolites with average postnatal weight and BMI z-scores among male offspring [50] - Some DEHP metabolites during the third trimester significantly related to weight gain rates from 6 to 12 months of age among boys, but DEHP levels during the second trimester positively related to weight gain rates at 6, 12, and 24 months old among boys [50] - Among girls, significant negative relationship between DEHP during the first trimester and weight gain rates, but no significant associations between DEHP and its metabolites and weight gain rates at 12 and 24 months [50] - DEHP exposure causes reduced fetal weight and crown-rump length, in a gender-independent manner [51] - Inverse association between urinary ∑DEHP metabolites levels and fetal growth [52] ***Neurocognitive outcomes:*** conflicting findings, with sex differences - Postnatal exposure linked to a decline in IQ scores [36,54] vs. no association between this exposure and psychomotor development [55] - Prenatal exposure was not associated with children’s IQ scores [36], neurodevelopment [56] (but sex plays a role of an effect modifier [56]), and risk of ASD [58] vs. inverse relationship between prenatal exposure and a child’s motor development [55] and psychomotor development (with increased odds of psychomotor delay) [57], with higher scores for autistic traits in boys but not girls [59] (these effects can be mitigated by folic acid supplementation [59])
**Organophosphates**	***Fetal outcomes:*** conflicting findings, with racial/ethnic differences - Prenatal exposure among black women correlated with decreased infant size at birth but no evidence of lower birth weight, birth length, or head circumference among whites or Hispanics [74] - No association between exposure measured at 28 weeks of gestation and birth weight, birth length, or abdominal or head circumference at delivery [75] - Exposure was associated with decreased fetal weight and length measured during mid-pregnancy, but not at delivery [76] ***Neurocognitive outcomes:*** conflicting findings but mostly negative effects, with sex differences - Prenatal exposure associated with poorer cognitive development [63] and Bayley scores in children [64] (particularly in boys [64]), and Neonatal Behavioral Neurological Assessment scores in neonates [70], increased number of abnormal primitive reflexes in neonates [68], poorer neonatal reflexes, attention skills, intellectual development [69], and development of ASD [71,72], with attenuating effect of folic acid supplementation in the first month of pregnancy on ASD in women exposed to organophosphates [73] - Inconsistent associations between prenatal exposure to organophosphates, pyrethroids, and carbamates and child development [65], and no significant association between IQ scores and prenatal exposure to organophosphates [66,67]
**Perfluoroalkyl substances (PFAS)** (including PFCAs, PFOA, PFSAs, PFHxS, PFOS, PFDA, and PFNA)	***Pregnancy course:*** conflicting findings with mostly negative effects - Strongly significant associations between serum concentrations of PFAS (PFDA and especially, PFNA) and miscarriage, and almost significant association with PFHxS exposure, while several studies reported no consistent link to miscarriage and stillbirths in a population with high PFOA exposure [79] - One of 12 studies found no association between PFAS exposure and thyroid dysfunction, while the rest showed altered thyroid hormone levels in either maternal blood or the umbilical cord after PFAS exposure, with the direction and magnitude of the associations remaining inconsistent throughout the studies [80] ***Fetal outcomes:*** mostly negative effects - Certain PFAS types partly reduce birth weight, birth length, and head circumference, and increase incidence of preterm birth, low birth weight, and small size for gestational age [84] - Higher early pregnancy PFAS concentrations associated with lower birth weight only among mothers whose early pregnancy dietary folate intake or plasma folate levels were below the 25th percentile [85] - Possible association between prenatal PFAS exposure and childhood obesity, as measured by BMI [81,82,83] ***Neurocognitive outcomes:*** mostly negative effects - No increase in ADHD prevalence in association with either exposure PFOS or PFOA, but stratified analyses suggested possible increased ADHD prevalence in association with PFAS exposure in girls, children from nulliparous women, and children from low-educated mothers (exposure was not exclusively prenatal) [86] - PFOA and PFOS exposure during pregnancy might be associated with ADHD in offspring, while prenatal PFOS and PFNA exposure might be associated with ASD in offspring [87] - PFAS exposure prenatally is not only associated with externalizing behaviors but also with internalizing behaviors such as general anxiety and major depressive disorder [88]

Abbreviations: bisphenol A—BPA; Autism Spectrum Disorder—ASD; blood pressure—BP; monoethyl phthalate—MEP; monobenzyl phthalate—MBzP; gestational diabetes mellitus—GDM; monoisobutyl phthalate—MiBP; monohydroxyisobutyl phthalate—MHiBP; monocarboxynonyl phthalate—MCNP; mono-n-butyl phthalate—MBP; monohydroxybutyl phthalate—MHBP; bis (2-ethylhexyl) phthalate/di (2-ethylhexyl) phthalate—DEHP; perfluoroalkyl substances—PFAS; perfluoroalkyl carboxylic acids—PFCAs; perfluorooctanoic acid—PFOA; perfluoroalkyl sulfonic acids—PFSAs; perfluorohexane sulfonic acid—PFHxS; perfluorooctane sulfonate—PFOS; perfluorodecanoic acid—PFDA; perfluorononanoic acid—PFNA.

## Data Availability

No new data were created.
